# *In Vitro* Effect of the Synthetic cal14.1a Conotoxin, Derived from *Conus californicus*, on the Human Parasite *Toxoplasma gondii*

**DOI:** 10.3390/md14040066

**Published:** 2016-04-08

**Authors:** Marco A. De León-Nava, Eunice Romero-Núñez, Angélica Luna-Nophal, Johanna Bernáldez-Sarabia, Liliana N. Sánchez-Campos, Alexei F. Licea-Navarro, Jorge Morales-Montor, Saé Muñiz-Hernández

**Affiliations:** 1 Departamento de Innovación Biomédica, Centro de Investigación Científica y de Educación Superior de Ensenada (CICESE), Baja California, C.P. 22860, Mexico; madeleon@cicese.mx (M.A.D.L.-N.); jbernald@cicese.edu.mx (J.B.-S.); lsanchez@cicese.mx (L.N.S.-C.); alicea@cicese.mx (A.F.L.-N.); 2 Subdirección de Investigación Básica, Instituto Nacional de Cancerología, Secretaría de Salud, San Fernando No. 22, Col. Sección XVI, Tlalpan, Ciudad de Mexico, C.P. 14080, Mexico; eunice.romero@gmail.com; 3 Departamento de Inmunología, Instituto de Investigaciones Biomédicas, Universidad Nacional Autónoma de Mexico; Ciudad de Mexico, C.P. 04510, Mexico; angitaluna87@gmail.com (A.L.-N.); jmontor66@biomedicas.unam.mx (J.M.-M.)

**Keywords:** conotoxin, *Conus californicus*, *Toxoplasma gondii*, antiparasitic toxin, parasite replication, host-cell invasion

## Abstract

Toxins that are secreted by cone snails are small peptides that are used to treat several diseases. However, their effects on parasites with human and veterinary significance are unknown. *Toxoplasma gondii* is an opportunistic parasite that affects approximately 30% of the world’s population and can be lethal in immunologically compromised individuals. The conventional treatment for this parasitic infection has remained the same since the 1950s, and its efficacy is limited to the acute phase of infection. These findings have necessitated the search for new drugs that specifically target *T. gondii*. We examined the effects of the synthetic toxin cal14.1a (s-cal14.1a) from *C. californicus* on the tachyzoite form of *T. gondii*. Our results indicate that, at micromolar concentrations, s-cal14.1a lowers viability and inhibits host cell invasion (by 50% and 61%, respectively) on exposure to extracellular parasites. Further, intracellular replication decreased significantly while viability of the host cell was unaffected. Our study is the first report on the antiparasitic activity of a synthetic toxin of *C. californicus*.

## 1. Introduction

*Toxoplasma gondii* is one of the most widely distributed parasites and it can infect any warm-blooded vertebrate. Host cell invasion by this parasite is a sequential process that involves several proteins from Apicomplexa specific secretory organelles [1,2] and parasite motility mechanisms that are based on its subpellicular cytoskeleton [3,4]. In host cells, Toxoplasma tachyzoites proliferate actively inside parasitophorous vacuoles (PVs), which are formed at the time of invasion from host cellular membrane components and parasite-secreted products [5,6]. Eventually, and in natural infections, PVs become tissue cysts that initiate the chronic phase of infection [7].

Toxoplasmosis is a congenital or acquired infectious disease that is induced by *T. gondii*. In immunocompetent human hosts, it is asymptomatic, but in immune-compromised persons, it can trigger certain infection-related pathologies, such as anophthalmia, blindness, hepatomegaly, encephalitis, and death [8,9]. Several reports indicate that approximately 80% of mortalities in HIV patients, and a high percentage of abortions are attributed to toxoplasma infection [10].

Since 1950, the most common treatment for toxoplasmosis has been the combination of two drugs that inhibit pyrimidine biosynthesis in the parasite: sulfadiazine (inhibitor of dihydropteroate) and pyrimethamine (inhibitor of dihydrofolate reductase) [8,11]. Unfortunately, this therapy has had limited efficacy, primarily for two reasons: (1) patient intolerance and adverse effects and (2) intrinsic parasitic factors that affect resistance and low susceptibility [8]. Further, this combination therapy does not affect the intracellular tachyzoite or cystic form of the parasite. It is estimated that one out of every three persons worldwide are infected with *T. gondii*. Considering the limitations of current treatment options, the development of more efficacious drugs that limit or cure this infection is an important area of research. The tachyzoite is the mobile and highly invasive form of *T. gondii* and is also its intracellular obligate form. Studies have suggested that that it is possible to find tachyzoites in an extracellular form, as a free-living parasite, in body fluids, such as blood, semen, and breastmilk, of several natural hosts [7]. Toxoplasma tachyzoites can invade practically all nucleated cells. Its active invasion is accompanied by the sequential secretion of many parasitic components and is regulated by its motor mechanism [12]. This invasive capacity is critical in its dissemination and prevalence in its natural host.

Molecules from natural origins (such as toxins from plants and sea animals) have emerged as notable therapeutic alternatives in several diseases [13,14]. For example, marine sources of such molecules include algae, sponges, corals, and snails. Several molecules have been extracted from these organisms for clinical and medical research applications, primarily because they have a wide array of unique structures, facilitating the discovery of new active compounds with a broad range of specific activity [14,15]. There are over 700 species of venomous marine snails that synthetize peptide toxins, most of which are distributed throughout tropical and subtropical waters [13,16]. *Conus californicus* is a marine snail species that is found in the eastern Pacific template (native of California, USA; Ensenada, Baja California, Mexico) [17]. Molecular studies have established that *C. californicus* is a phylogenetically distant species from other marine snails [18,19]. 

Conotoxins are typically 10–40 amino acids in length and are cysteine-rich [16]. They target a wide range of receptors and ion channels with high potency and selectivity [13,16]; many such compounds are used as pharmacological agents and have significant diagnostic and therapeutic potential. Conotoxins are currently used to treat chronic pain and myocardial infarction [16,20,21], but their therapeutic efficacy as antiparasitic agents has not been tested. Twenty-seven gene superfamilies that are related to the signal sequence of conotoxin precursors and 12 pharmacological families of conotoxins have been characterized from cone snails (http://www.conoserver.org). Conotoxins have two remarkable molecular features: (1) they are members of a family of peptides that target various types of membrane proteins; and (2) the structure and specificity of conotoxins depend on the feeding habits of the snails [17,18]. Little information exists about the functional characteristics of conotoxins in *C. californicus*. In a previous study, 15 peptides were purified and analyzed biochemically, seven of which were classified as members of a new gene superfamily (named J2). One such compound was cal14.1a [18].

Based on these findings, we determined the *in vitro* effects of the conotoxin s-cal14.1a on the survival, invasion, and intracellular proliferation of extracellular tachyzoites of *T. gondii*.

## 2. Results

### 2.1. Decreased Extracellular Viability of Tachyzoites Induced by s-cal14.1a in Vitro

**Figure 1:**
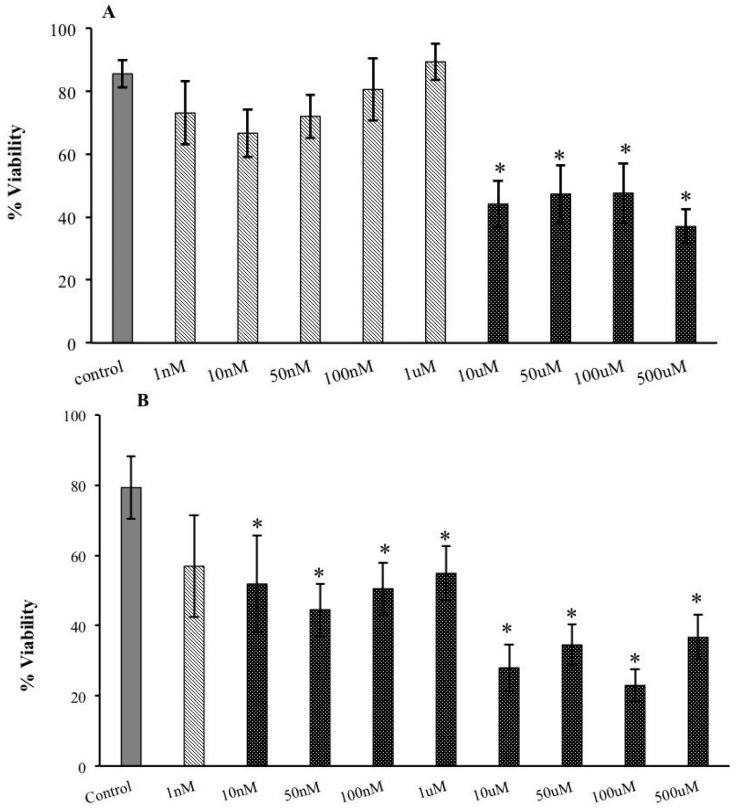
Effects of s-cal14.1a on extracellular tachyzoites of *T. gondii.* (**A**) Viability was measured by exclusion technique; purified tachyzoites were exposed for 30 min to various concentrations of s-cal14.1a. Inhibition of viability was observed between 10 μM and 500 μM; (**B**) Two-hour exposure to s-cal14.1a induced high mortality in extracellular tachyzoites. (*) indicates significant differences between control and treatment. *p* ≤ 0.001.

Exposure of extracellular tachyzoites to increasing nanomolar concentrations of s-cal14.1a for 30 min had little to no effect on parasite viability (Figure 1A). However, in the micromolar range, viability decreased significantly (50%), starting at 10 μM, compared with extracellular tachyzoites that were not exposed to toxins (Figure 1A). Extracellular tachyzoite viability fell significantly on exposure for 2 h (Figure 1B)—viability declined by approximately 35% starting at 10 nM to over 60% at all micromolar concentrations of the conotoxin (Figure 1B).

### 2.2. Invasivity of Tachyzoies of T. gondii after Exposure to s-cal14.1a

**Figure 2:**
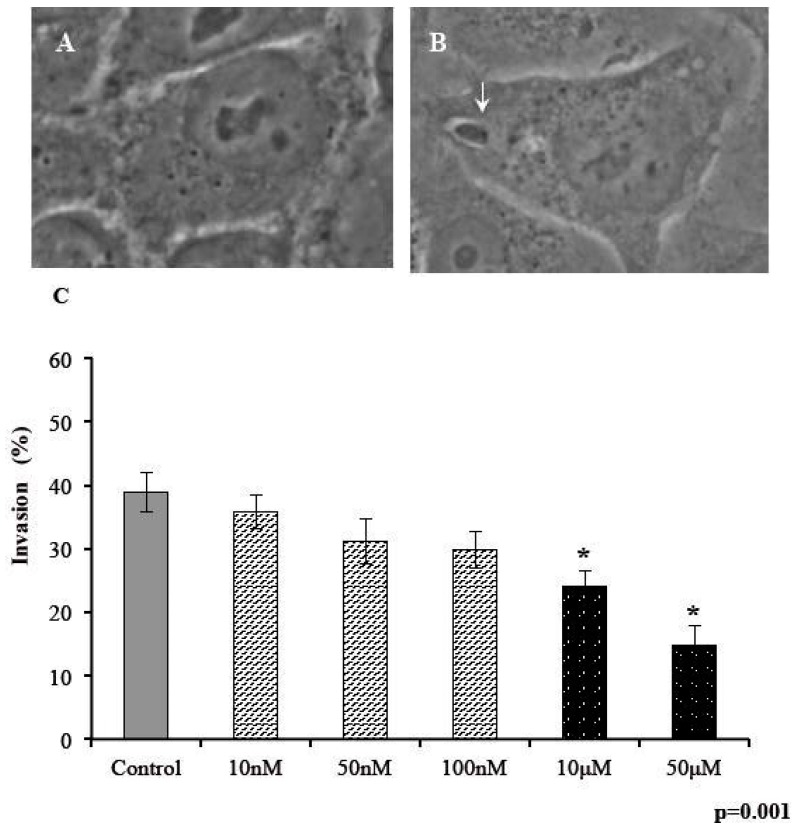
Invasion of *T. gondii* tachyzoites decreases on exposure to s-cal14.1a. Phase-contrast micrograph of (**A**) uninvaded cell and (**B**) invaded cell with a single parasite. (**C**) Percentage invasion of tachyzoites pretreated with s-cal14.1a for 30 min. (*) indicates significant differences between control and treatment. *p* = 0.001. Arrow indicates a single intracellular parasite.

We examined the ability of parasites to penetrate human cells after 30 min of exposure to s-cal14.1a at five concentrations. The number of infected cells was plotted as a percentage of invasion (Figure 2). We defined infected cells as all cells that harbored at least 1 PV that contained a parasite in the cytoplasm (Figure 2B). Similar to the results above, extracellular exposure of tachyzoites to s-cal14.1a in the nanomolar range did not induce any differences in invasiveness compared with the control (Figure 2C), but at micromolar concentrations, invasive capacity differed significantly (Figure 2C).

### 2.3. Proliferation of T. gondii Tachyzoites after Exposure to s-cal14.1a

**Figure 3:**
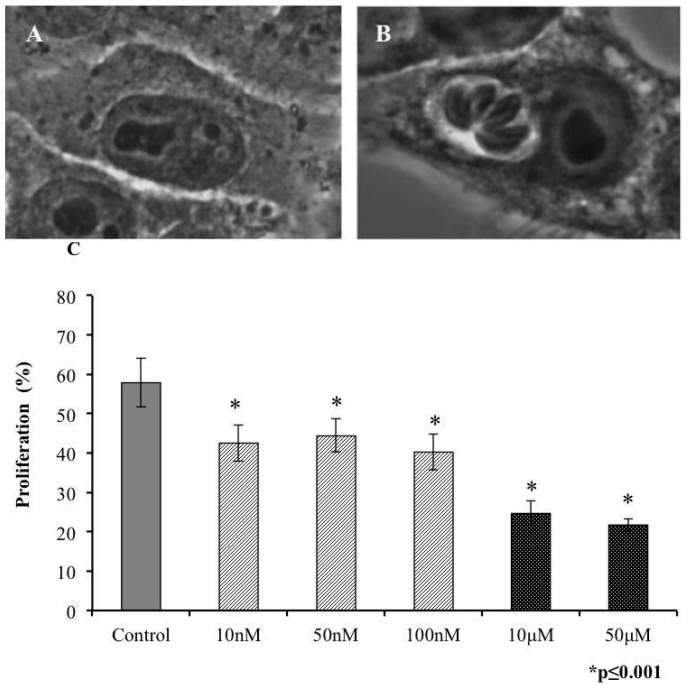
Proliferation of *T. gondii* tachyzoites decreases on s-cal14.1a exposure. Phase-contrast micrograph of (**A**) control cell and (**B**) proliferating PV in a single cell. (**C**) Percentage of proliferating PVs in tachyzoites pretreated with s-cal14.1a for 30 min. (*) indicates significant differences between control and treatment. *p* ≤ 0.001.

On entering the host cell, a tachyzoite begins its replication. *In vitro* studies have indicated that each replication cycle comprises approximately 6 h. Thus, we analyzed the effect of our conotoxin on parasite replication. Extracellular tachyzoites, pretreated with toxins, were incubated with HEp-2 cells for 24 h to permit at least three replications cycles. We considered proliferated cells as those that contained at least eight parasites in PVs (Figure 3B). Proliferation differed significantly at all concentrations *versus* the control (Figure 3C)—the most notable effect was observed at 10 and 50 μM, at which only 20% of parasites replicated.

### 2.4. Presence of s-cal14.1a Toxin in Culture Medium Inhibits Proliferation of Tachyzoites

**Figure 4:**
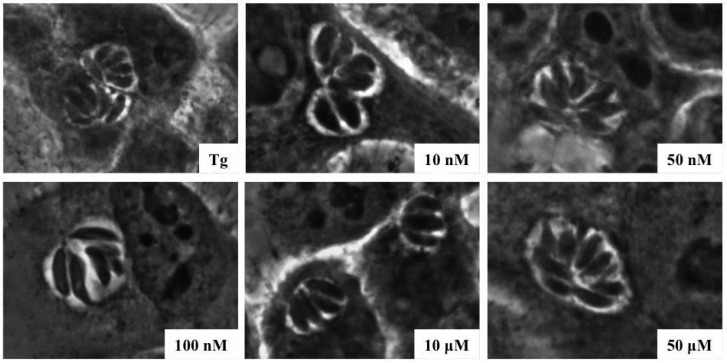
s-cal14.1a in culture medium inhibits proliferation of tachyzoites. Phase-contrast micrographs of the morphology of intracellular replication of toxoplasma after exposure to s-cal14.1a. Tg: Normal tachyzoite replication. The remaining five micrographs indicate the s-cal14.1a concentration used to impede parasitic replication.

In natural infections, tachyzoites must be inside their host cells to replicate or differentiate. To analyze the effects of s-cal14.1a on intracellular replication after tachyzoites established their PVs in cells, we permitted the tachyzoites to invade their host cells under ideal culture conditions. Then, after 1 h, we added toxins at several concentrations, and the cells were incubated for another 24 h. Figure 4 shows micrographs of the intracellular replication of tachyzoites when the conotoxin was added to HEp-2 cells after invasion, in which parasitic proliferation can be observed independently of conotoxin concentration in the culture medium (Figure 4).

**Figure 5:**
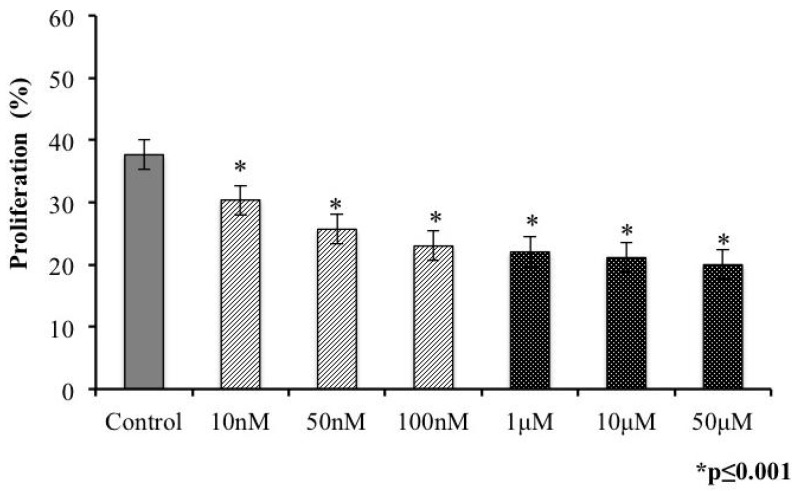
Exposure of cellular microenvironment to s-cal14.1a partially inhibits proliferation. Graph shows the effects of s-cal14.1a on replication of tachyzoites on exposure after infection. (*) Indicates significant differences between control and treatment. *p* ≤ 0.001.

We measured the percentage of proliferated cells—*i.e.*, those with at least eight parasites per inner PV. Proliferation diminished gradually, leveling off at micromolar concentrations (Figure 5). In the control samples, nearly half of the cell monolayers in the culture contained proliferating PVs (Figure 5), and the differences in replication at all concentrations were significant (Figure 5, asterisks). In our experimental conditions, proliferation was inhibited by approximately 50% with respect to the control. As shown in Figure 5, the inhibition of proliferation had a similar pattern as that in extracellular tachyzoites that were treated with s-cal14.1a (see Figure 5 *vs.* Figure 3C).

### 2.5. Cytotoxic Effects of s-cal14.1a on Tachyzoite Host Cells

**Figure 6:**
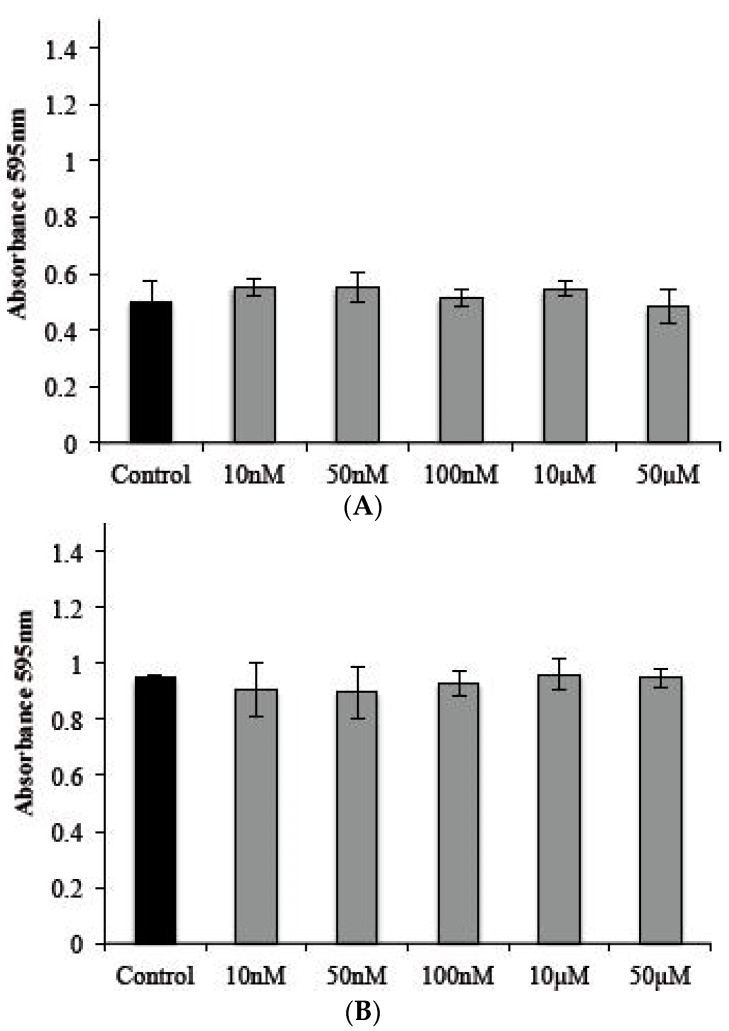
Effect of s-cal14.1a on HEp-2 cells. A total of 3 × 10^4^ cells per well were treated with s-cal14.1a (**A**) by 2 h and (**B**) by 24 h; these npt show any differences compared with their control. Control cells were cultured in EMEM medium without toxin.

We analyzed the effects of s-cal14.1a on a cell line that was used as host cells for *T. gondii* tachyzoites, at the same concentrations as in the previous section. There was no cytotoxic effect after 2 or 24 h compared with a culture that lacked the toxin (Figure 6A,B).

## 3. Discussion

Several antimicrobial peptides have been isolated from a wide range of organisms and characterized. However, less than 0.1% of them have been pharmacologically tested, and even fewer are being used in clinical and preclinical studies [22,23].

In this study, we have described the *in vitro* effects of a synthetic peptide, isolated from *C. californicus*. At increasing concentrations of the conotoxin, parasite viability was significantly inhibited under extracellular conditions. This decrease was most notable with s-cal14.1a after long incubation times. The duration of exposure to a conotoxin is an important factor in the survival of extracellular tachyzoites. It is possible that during the acute phase of infection, s-cal14.1a remains in circulation long enough to gain access to a sufficient number of tachyzoites in the host. *Toxoplasma* is a reemergent parasite; thus, this toxin could have effects during its reemerging phases.

Tachyzoite activity is important in establishing its infection in host cells—primarily nonphagocytic cells, which constitute the majority of cells in any organism. We found that s-cal14.1a inhibits invasion and proliferation, even at nanomolar concentrations. Our data correlate with other studies that have tested small peptides with various origins. In these reports, the viability of extracellular tachyzoites of *T. gondii* and its ability to penetrate cells declined [24,25,26]. In contrast, we did not observe any aggregation of parasites on the plasma membrane of host cells, even at toxin concentrations in the same range of compounds that were used by other groups [24,26], suggesting that the peptide from *C. californicus* has unique features [18] and a different mechanism of action that affects invasion and proliferation when extracellular tachyzoites are exposed directly. 

Certain extracts of marine seaweeds (in addition to peptides and toxins) have been used as antiprotozoal compounds with conflicting results [15,27]. One study that examined organic extracts of French marine seaweeds on Plasmodium falciparum—a parasite that is highly related to toxoplasma—found that extracts that were obtained with nonpolar solvents had more activity *versus* polar solvents; nearly 40% of them had effects against plasmodium [27]. In another report, nonpolar fractions of a brown seaweed had greater efficacy against parasites that were not related to toxoplasma [15]. Thus, the intrinsic characteristic of compounds and toxins determine their parasiticidal effects. At nanomolar concentrations of s-cal14.1a, we did not observe any notable effects on extracellular parasites. Consistent with these data, s-cal14.1a did not affect invasion. However, extracellular exposure to s-cal14.1a toxin altered the proliferation of tachyzoites (30% decrease in proliferation with respect to untreated tachyzoites). In contrast, micromolar concentrations of s-cal14.1a had greater effects on the viability, invasiveness, and proliferation of toxoplasma. Considering that conventional treatments for toxoplasmosis are applied over a wide range of concentrations [15], the efficacy of micromolar doses of s-cal14.1a could be an advantage.

In natural infections, tachyzoites established themselves in host cells to start the chronic phase of toxoplasmosis, which includes their replication and differentiation toward bradyzoites [7,28]. However, in toxoplasma infections, there are no drugs that are effective when tachyzoites localize to cytoplasmic PVs; all antitoxoplasmic drugs act solely on extracellular tachyzoites [8]. We used the most virulent strain of toxoplasma (RH, lethal dose of 100% with 1 viable parasite [29]) and found that the addition of s-cal14.1a to the culture medium of host cells after invasion affects its intracellular proliferation, resulting in a minor percentage of proliferating vacuoles. The principal advantage of this conotoxin might be that it has activity within cells against intracellular parasites, altering their establishment of infection. We hypothesize that s-cal14.1a crosses the apical membrane of the host cell and PV and parasite membranes, thus disrupting the replication machinery of the parasite, based on our observation that parasite proliferation declined without the the host cell being affected. Additional experiments are needed to test this hypothesis, for example, by administering radioactive toxin to cells. Perhaps most significantly, s-cal14.1a is not toxic to normal cells. Several studies have examined toxins that are related to the genus Conus, based on their ability to selectively target certain subtypes of ion channels and receptors in membranes [13,30]. Thus, we wish to extend our studies beyond the applications that we have described and test their effects in an animal model of experimental toxoplasmosis. The broad distribution of receptors and channels over a wide range of eukaryotic and prokaryotic organisms, including protozoa, such as toxoplasma, render the toxins a source of novel activities. Biological processes in protozoa are associated with the activity of various ion channels. Specifically, calcium channels mediate host cell invasion by *T. gondii* [31,32], and potassium channels and acetylcholine receptors participate in intracellular replication [33]. Although the involvement of conotoxins, such as s-cal14.1a, with regard to acetylcholine receptors has been hypothesized, their mechanism of action against *T. gondii* is unknown, although its antiparasitic activity is evident. s-cal14.1a has been reported to demonstrate activity in lung cancer cell lines S that express α5-n-acetylcholine receptors [34].

cal14.1a belongs to a family of highly similar conotoxins, with sequence identities ranging from 41% to 94% and harboring the conserved Cys #XIV framework. However, peptides in the J superfamily with the same Cys pattern [35] diverged from the cal14 family in their signal sequence. Instead, the cal14 family has been related to lt14a (Supplementary Figure 1A,B) in the J2 superfamily, based on their shared C-C-C-C framework and similarities in signal sequences [18]. The *C. litteratus* peptide has recently been reported to target neuronal nicotinic acetylcholine receptors and participate in the nAChR pathway with Ca^2+^ signaling. Lt14a suppresses intracellular Ca^2+^ i by blocking nAChR [36]. Ca^2+^ signaling is a vital pathway in *T. gondii*, in which there is a correlation between Ca^2+^ concentration and virulence traits [37].

Further study is needed to define the parasitic activities of s-cal14.1a. We have presented novel findings that it has effects in free and intracellular tachyzoites. Also, this conotoxin alters the invasiveness and proliferation of tachyzoites. It is likely that s-cal14.1a will decrease morbidity and mortality in a mouse model of acute toxoplasmosis if it is used as a prophylactic or curative treatment. Further, it can be combined with conventional drugs for toxoplasmosis. The specific target of s-cal14.1a in toxoplasma tachyzoites, however, must be identified. Future studies should address these points, and determine the value of conotoxins as antitoxoplasmic drugs.

## 4. Materials and Methods

All material and reagents were purchased from J.T. Baker Co. (J.T. Baker Chemical Company, Phillipsburg, NJ, USA), unless otherwise indicated.

### 4.1. Ethics Statement

Animal care and experimentation practices at the Instituto Nacional de Cancerología were constantly monitored by the Institute’s Animal Care and Use Committee, adhering to official Mexican regulations (NOM 062-ZOO-1999). Mexican regulations were implemented in strict accordance with the recommendations per the Guide for the Care and Use of Laboratory Animals of the National Institute of Health (NIH, USA) to ensure compliance with established international regulations and guidelines. Efforts were always made to minimize animal suffering. Male Balb/c AnN (H2-d) inbred mice, obtained from Harlan (Mexico City), were used in all of the experiments. Animals were housed in the animal care facilities of Instituto Nacional de Cancerología, under controlled temperature (22 °C) and 12-h dark-light cycles, with the lights on between 07:00 and 19:00. They were fed Purina Diet 5015 (Purina, St. Louis, MO, USA) and tap water ad libitum. The RH strain of *T. gondii* was used for infection. A total of 50,000 tachyzoites were suspended in 0.1 mL sterile phosphate-buffered saline (pH 7.2) and carefully injected intraperitoneally into 6–8-week-old male mice using a 0.27-gauge needle. Infected mice were housed in separated cages (2 each) in the same room of the animal facility. At 4–5 days of infection, the mice were euthanized rapidly by cervical dislocation.

### 4.2. Maintenance and Purification of T. gondii Tachyzoites

RH strain tachyzoites were maintained by intraperitoneal passage in male Balb/c mice. Parasites were recovered from peritoneal exudates, washed with 1× PBS (138 mM NaCl, 1.1 mM K_2_PO_4_, 0.1 mM Na_2_HPO_4_, and 2.7 mM KCl, pH 7.2), and purified by filtration through 5-μm-pore polycarbonate membranes (Millipore Co., Bedford, MA, USA).

### 4.3. Cell Culture

The HEp-2 cell line, originally derived from an epidermoid carcinoma of the larynx (ATCC-CCL 23), was used as host cells for parasite invasion and proliferation. HEp-2 cells were maintained in minimum essential medium MEM) (GIBCO, Thermo Fisher Scientific, New York, NY, USA), supplemented with 10% fetal bovine serum (FBS, ATCC, Manassas, VA, USA), under a 5% CO_2_ atmosphere at 37 °C.

### 4.4. Conotoxin s-cal14.1a

Conotoxin s-cal14.1a was first isolated from *Conus californicus*, named and reported by Biggs *et al.* 2010 (Genebank Fj959129) [18]. In this study, we used a synthetic form that was obtained from Ontores Biotechnologies Co., Ltd (Hangzhou, Zhejiang, China).

### 4.5. Exposure of Extracellular Tachyzoites to Conotoxin

Purified parasites were placed in non-supplemented MEM to a final concentration of 6 × 10^6^ tachyzoites per 100 μL, exposed to several conotoxin concentrations (10 nM, 50 nM, 100 nM, 1μM, 10 μM, and 50 μM; s-cal14.1a was resuspended in 1× PBS) for 30 min and 2 h at room temperature (RT), and slowly agitated. The ratio between live and dead tachyzoites was measured by exclusion technique with trypan blue; 500 parasites were counted in each assay under an optical microscope (63×/1.4 objective, AxioOberve Microscope, Carl Zeiss Mexico).

### 4.6. Infection of HEp-2 with RH Strain

HEp-2 cells were grown on sterile coverslips in MEM that was supplemented with 10% FBS for 24 h until 80% confluence. Cells were exposed to parasites at a parasite: host cell ratio of 5:1, incubated for 2 h, and washed with PBS to discard extracellular parasites.

For the invasion assays, infection was stopped after the monolayers were washed on addition of 3.7% formaldehyde for 30 min at RT with agitation. Then, the samples were washed with 1× PBS and stained with hematoxylin and eosin.

The samples were mounted in glycerol: 1× PBS and analyzed by mean optic microscopy (100×/1.2 objective; AxioOberve Microscope, Carl Zeiss, Mexico). At least 300 invaded cells were counted—*i.e.*, those that contained one or more PVs in the cytoplasm.

In the proliferation assays, after extracellular parasites were eliminated, fresh supplemented medium was added to the monolayers and incubated for additional for 24 h. Next, the infection was stopped with 3.7% formaldehyde as in the invasion assays. Proliferation was measured in cells that harbored at least 8 parasites in PVs, for which 300 cells were counted. All assays were performed independently in triplicate. 

### 4.7. Effects of Conotoxin on Invaded Cell Culture

HEp-2 cell monolayers were invaded with toxoplasma tachyzoites at a 5:1 ratio for 2 h. Extracellular parasites were eliminated by rapid washing, and several concentrations of conotoxin (1, 10, and 100 nM and 1, 10, and 50 μM) were added. Conotoxin was resuspended in MEM that was supplemented with 10% FBS and incubated for an additional 24 h. Proliferating cells were measured as in the proliferation assays.

### 4.8. Cytotoxic Effect of s-cal14.1a on Tachyzoites Host Cells.

The cytotoxic effects of s-cal14.1a were measured in Hep-2 cells. Briefly, individual wells of a 96-well plate (Nunc, Wiesbaden, Germany) were inoculated with 200 μL of culture medium that contained 3 × 10^3^ cells.

The plates were incubated for 24 h to achieve 60%–70% confluence. Cell monolayers were exposed for 2 and 24 h to increasing concentrations of s-cal14.1, and cell viability was measured by crystal violet staining. After exposure to s-cal14.1a, the samples were washed with 1× PBS and fixed with 10% formaldehyde. After crystal violet staining for 4 h, the monolayers were washed with acetic acid, and absorbance was measured at 495 nm. DMSO (10%) was used as a positive control of cell toxicity. Cells in EMEM alone were used as a control group.

### 4.9. Statistical Analysis

For descriptive purposes, continuous variables were summarized as arithmetic means and standard deviations (SDs). Inferential comparisons were made using student’s *t*-test or Mann-Whitney *U*-test, depending on the data distribution (normal and non-normal, respectively), as determined by Kolmogorov-Smirnov test. Post hoc analyses were conducted using Tukey’s test. Statistical significance was determined at a *P*-value of 0.05 by a two-tailed test. SPSS version 20 (SPSS Inc., Chicago, IL, USA) was used for the data analysis.

All assays were performed in triplicate at least 3 times.

## 5. Conclusions

We have demonstrated that the s-cal14.1a conotoxin has significant effects on toxoplasma tachyzoites in their extracellular form. s-cal14.1a can “reach” intracellular parasites, despite their natural barriers (such as their apical cell membrane and pellicle). In this novel study, we have determined that a small peptide of marine origin has parasiticidal activity against *Toxoplasma gondii*. Further studies should be performed to determine its efficacy in a complete disease model, such as acute murine toxoplasmosis.

## Supplementary Materials

The following are available online at http://www.mdpi.com/1660-3397/14/3/66/s1. Figure S1: Comparison of some predicted J2-superfamily members.
