# Supplementary material for: In Vitro Effect of the Synthetic cal14.1a Conotoxin, Derived from Conus californicus, on the Human Parasite Toxoplasma gondii
Source: Mar Drugs. 2016 Apr 8;14(4):66. doi: 10.3390/md14040066 (PMC4849070; doi:10.3390/md14040066)
Supplement: Supplementary file 1 [file marinedrugs-14-00066-s001.pdf]

Supplementary Materials: In Vitro Effect of the Synthetic cal14.1a Conotoxin, Derived from *Conus californicus*, on the Human Parasite *Toxoplasma gondii*

Marco A. De León-Nava , Eunice Romero-Núñez , Angélica Luna-Nophal, Johanna Bernáldez-Sarabia, Liliana N. Sánchez-Campos, Alexei F. Licea-Navarro, Jorge Morales-Montor and Saé Muñiz-Hernández

(**A**)

```
cal14.1a    GDCPPWCVGA--RCRAEKC
             .***.*..:   *    *
lt14a       -MCPPLCKPS---CTN--C
```

(**B**)

cal14.1a	GD	C	PPW	C	VGAR	C	RAEK	C	
cal14.1b	GD	C	PPW	C	VGAR	C	RAGK	C	
cal14.2a	RE	C	PPW	C	PTSH	C	NAGT	C	
cal14.2b	RE	C	PPR	C	PTSH	C	NAGT	C	
cal14.2c	RD	C	PPW	C	PTSH	C	NAGT	C	
cal14.3	RQ	C	PPW	C	SGEP	C	RKGT	C	
lt14a	M	C	PPL	C	KPS	C	TN	C	*

Figure S1. Comparison of some predicted J2-superfamily members. (**A**) Alignment of cal14.1a and lt14a by CLUSTAL W2, pairwise sequence alignment (edited). (**B**) Multiple alignment of several conotoxins belonging J2 superfamily. The asterisk indicated an amidated C-terminus. The gray column indicated the conserved cysteine residues.
